# Association Between Fish Consumption and Muscle Mass and Function in Middle-Age and Older Adults

**DOI:** 10.3389/fnut.2021.746880

**Published:** 2021-12-13

**Authors:** Maha H. Alhussain, Moodi Mathel ALshammari

**Affiliations:** Department of Food Science and Nutrition, College of Food and Agriculture Sciences, King Saud University, Riyadh, Saudi Arabia

**Keywords:** sarcopenia, fish, protein, omega-3 fatty acids, muscle mass, muscle function

## Abstract

**Background:** Sarcopenia, the age-related loss of skeletal muscle mass and function, represents a crucial risk factor for disability and mortality. Increasing intake of some nutrients, particularly protein and omega-3 fatty acids seems to be a promising strategy to augment muscle mass and function.

**Objective:** The purpose of this study was to assess the beneficial effects of fish consumption on muscle mass and function among middle-age and older adults.

**Methods:** Twenty-two adults aged 50–85 years participated in this study. Participants were asked to consume 150–170-g of fish for lunch twice a week for a 10-week period. During that period, participants were asked to maintain their normal diet and physical activity. Outcome measures included anthropometry, muscle mass, and muscle function. All these measures were assessed at baseline, week 5, and week 10. Repeated-measures analysis of variance was used to analyze statistical significance.

**Results:** Consuming fish twice a week for 10 weeks significantly increased the skeletal muscle mass and appendicular lean mass divided by height squared (ALM/h^2^) (*p* < 0.01). Handgrip strength and gait speed <0.8 m/s were also improved (*p* < 0.01) at week 10 compared with that at baseline.

**Discussion:** Consuming fish seems to improve muscle mass and function and may slow sarcopenia progression in middle-age and older adults.

## Introduction

The global population aged 60 years and above is growing faster than all younger age groups. In 2017, they numbered 962 million and is expected to increase to 2.1 and 3.1 billion by 2050 and 2100, respectively (1). Population aging is projected to have profound impacts on societies, underscoring the economic consequences that the healthcare sector is likely to face in many countries. A leading health issue in aging individuals is sarcopenia, which has significant clinical implications (2). The term “sarcopenia” was first defined as an age-related syndrome characterized by a progressive loss of skeletal muscle mass (SMM) (3). Recently, various expert groups from around the world have published consensus definitions and recommended using the presence of low muscle mass in combination with poor muscle function (muscle strength and physical performance) for diagnosing sarcopenia (4–6).

Interest in preventing and managing sarcopenia by improving muscle health is growing. Numerous studies have identified modifiable risk factors for sarcopenia, including diet (7, 8). Poor diet and nutrition status among older adults are often cited (9–11). Adopting a healthy diet can be an effective strategy to promote healthy aging. Seafood, including fish, is considered a part of a healthy diet, and consumption of at least 8 ounces of seafood per week is recommended (12). Furthermore, the recommended fish consumption is at least twice a week (13). Fish are a high-quality source of protein and omega-3 fatty acids, which are the dominant polyunsaturated fatty acids of fish oil. Fish are also rich in vitamins, including vitamin D. Protein and vitamin D are nutrients that have been consistently linked to sarcopenia determinants—muscle mass and muscle function (8). Indeed, protein is recognized as a key nutrient for better health among older adults (14). It provides amino acids that are required for muscle protein synthesis and acts as an anabolic stimulus directly effecting protein synthesis (8, 15). Vitamin D enhances muscle protein synthesis and boosts strength and balance (16, 17). However, the mechanisms by which vitamin D enhances muscle mass and function are not fully understood (8). In addition to protein and vitamin D, omega-3 fatty acids are suggested to be related to sarcopenia (18–20). Sarcopenia is proposed as an inflammatory state, and omega-3 fatty acids could be potent anti-inflammatory agents (18). In addition, fish contain vitamin E, which could be beneficial for maintaining muscle health. Considering the health benefits of the nutrients that fish contain, we hypothesized that nutritional intervention with fish would improve muscle mass and function. Therefore, this study was conducted to examine the beneficial effects of fish (i.e., red sea bass) consumption for 10 weeks on muscle mass and function among free-living middle-age and older adults.

## Methods

### Study Participants

Twenty-two middle-age and older adults (eight men and 14 women), aged 50–85 years participated in this study. They were recruited via advertisements on social media and posters placed in several health centers in Riyadh, Saudi Arabia. Potential participants were excluded if they had any of the following conditions: disabilities, mental health issues, liver or kidney diseases, fish allergy, and body weight that was unstable in the last 3 months (≥ ± 3 kg).

The study was conducted at two health centers in two regions, east and north of Riyadh, between December 2018 and May 2019. The participants voluntarily signed consent forms before inclusion in the study.

### Calculation of Sample Size

A study (21) has demonstrated that muscle mass increases after an 8-week intervention with whey protein supplementation (from 59.6 ± 5.2 to 62.8 ± 5.2 kg). Therefore, 24 individuals were required to detect a difference in muscle mass with a power of 80% at a significance level of 0.05 using G^*^Power (Heinrich-Heine-Universität Düsseldorf, Brunsbüttel, Germany).

### Study Design and Procedure

A within-subject experimental design was used in this study. Potential participants were screened using a general health questionnaire to assess their eligibility. They also completed the Mini-Nutritional Assessment Scale–Short Form (MNA-SF) to evaluate their nutritional status (22). Eligible participants were enrolled in a 10-week intervention. During the intervention period, participants were asked to consumed a filet portion of fish twice a week, with 3 days apart, and were asked to maintain their habitual diet and perform their normal daily physical activities. The investigator confirmed that all participants followed the instructions through weekly phone contact. Outcome measures including anthropometry (body weight; height; body mass index, BMI; waist circumference; hip circumference and waist-to-hip ratio), body composition (body fat, BF%; fat mass, FM; and SMM), and sarcopenia parameters (SMM index, SMI%; appendicular muscle mass divided by height squared, ALM/h^2^; handgrip strength and gait speed) were assessed by a trained staff at baseline, week 5, and week 10 of the intervention.

### Dietary Intake

The participants were instructed to complete a 3-day food record in which to record their food intake over 3 days (2 weekdays and 1 weekend day) to estimate their habitual dietary intake. Detailed instructions on how to complete the food diary were provided to each participant by a trained staff using semi-quantitative household measures. The diary was entered and analyzed using Food Processor (version 11.6, ESHA Research, Inc., Salem, OR). The habitual dietary intakes of the participants were compared with the 2015–2020 (8th edition) Dietary Guidelines (23). A filet portion of red sea bass was added to the participants habitual diet to calculate their intake with the additional fish intake. The total energy intake and macronutrients (carbohydrates, proteins, and fat) as grams and percentage contribution of energy were computed. Vitamins D and E were also assessed.

### Intervention

The participants were instructed to consume one filet portion (150–170g) of red sea bass fish (also known as the barramundi sea bass) with their habitual diet at lunch twice a week for 10 weeks. The red sea bass was chosen in this study because it has been cultured in many Asian countries, including Saudi Arabia (24), has a good market price, and is preferred by consumers due to its delicate and mild-flavored white meat (25). In the current study, fish were supplied by the National Aquaculture Group (NAQUA, Riyadh, Saudi Arabia), transported to Zero Fat Restaurant (Riyadh, Saudi Arabia) in two batches—the first one was one day before the start of the study and the second one was after 5 weeks of the intervention—and stored at −20°C. One portion of filet was cooked and packaged for each participant and then delivered to participants' homes at lunch time as scheduled. The restaurant was advised to cook each portion of fish with a piece of lemon for approximately 20 min in an oven at 200°C. The amount of fish given to the participants was based on the Dietary Guidelines for Americans (Dietary Guidelines Advisory Committee 2015) (26). The investigator contacted the participants during the intervention period to follow up with their compliance to fish consumption.

### Measurements

The following parameters were measured during the designated visits (baseline, week 5, and week 10) by the same investigator at the same time and under the same conditions according to the standard procedures. Body weight (kg) was measured to the nearest 0.1 kg using a digital scale. With the scale placed on a hard-flat surface and with the digital screen indicating zero, the participants were instructed to stand on the scale with both feet without shoes, wearing light clothes and with an empty bladder. Height was measured at inclusion, for the computation of BMI, barefoot to the nearest 0.1 cm using a stadiometer. BMI was calculated using the standard formula: body weight/height^2^ (kg/m^2^). The waist circumference was measured using a tape measure at the midpoint between the lower margin of the last palpable rib and the top of the iliac crest. The hip was measured at the widest portion of the buttocks. The waist-to-hip ratio was calculated as the ratio of the waist and hip circumferences.

Body composition was measured by bioelectric impedance analysis (BIA, Inbody 270, Cerritos, CA, USA). The participants stood on a balance scale in bare feet and held the conductive handles. After the 15-s measurement, the results were printed. The percentage of BF%, FM (kg), and SMM was obtained through body composition analysis.

SMI% was calculated by dividing the SMM by the body mass (SMM [kg]/body mass [kg] × 100) (27). ALM, which was defined as the sum of the lean masses of the arms and legs, was calculated using the following formula: ALM (kg)/ht^2^ (m^2^) (28).

Handgrip strength was assessed using the Jamar hand dynamometer (J00105, Lafayette Instrument Company, USA). The participants were instructed to stand with their arms and wrists stretched out at the sides of the body. Then, they were instructed to squeeze the handle as hard and as tightly as they could for 3–5 s. This measure was performed three times on each hand, with 60 s of recovery between each measurement. For the statistical analysis, the best value (in kg) among the repeated measurements was taken.

Physical performance was assessed using gait speed over a 4-m distance. The participants were instructed to walk at their usual speed with a static start without deceleration throughout a 4-meter straight line. Using a stopwatch, the time was recorded. Gait speed was expressed in meters per second (m/s) (29).

### Statistical Analysis

Statistical analyses were performed using Statistical Package for the Social Sciences (version 22; IBM Corp., Armonk, NY, USA). Data were checked for normality of distribution using the Kolmogorov–Smirnov test. Continuous data were presented as means ± standard deviations (SDs), and categorical data were presented as frequencies and proportions. The comparisons between the participants' habitual diet and DRI were performed using a paired *t*-test. One-way analysis of variance was performed to examine variations between the measured variables (anthropometry, body composition and sarcopenia parameters) across the study. Where significant main effects were determined, Fisher's least significant difference *post-hoc* test was conducted to determine pairwise differences between baseline, week 5, and week 10. *P*-values of < 0.05 were used to denote statistical significance.

## Results

**Figure 1:**
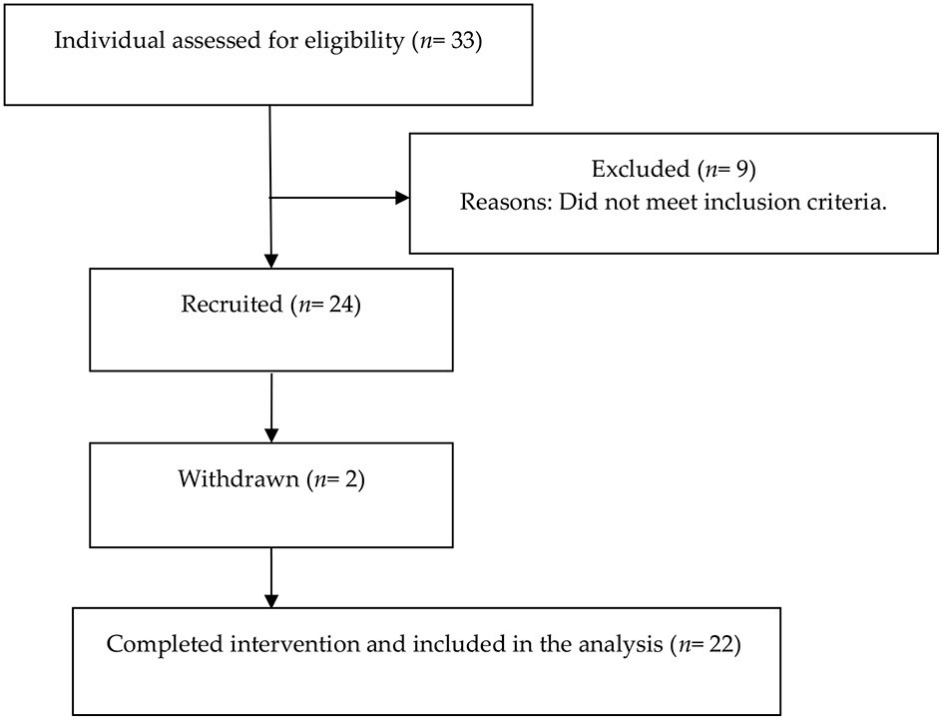
Flow chart diagram of the study participants.

A total 33 middle-age and old adults (13 men and 20 women) were initially screened for eligibility, nine of whom (five men and four women) were excluded as they did not meet the inclusion criteria. Twenty-four participants (eight men and 16 women) enrolled in the study. Two women withdrew before the end of the study because they moved away. The final study sample consisted of 22 participants (eight men and 14 women). The flow chart of the study participants is displayed in Figure 1.

### Baseline Characteristics

Table 1 shows the baseline general and clinical characteristics of the study participants. The mean age of the participants was 60.18 ± 9.0 years, based on the MNA-SF, the majority of the participants (91%) were non-malnourished.

**Table 1:** Baseline general and clinical characteristics of the study participants.

**Characteristic**	**Total (*n =* 22)**
Age (years)	60.18 ± 9.0
Height (cm)	160.61 ± 6.38
Body weight (kg)	64.25 ± 11.71
BMI (kg/m^2^)	25.97 ± 4.82
Waist (cm)	90.50 ± 10.15
Hip (cm)	102.28 ± 8.60
Waist–hip ratio	0.89 ± 0.09
BF %	39.16 ± 9.22
FM (kg)	25.81± 8.96
SMM (kg)	20.62 ± 3.87
SMI (%)	31.21 ± 4.68
ALM/ht^2^ (kg/m^2^)	6.05 ± 1.02
Handgrip strength (kg)	20.63 ±7.11
Gait speed <0.8 m/s	1.35 ± 0.78
Nutritional status (MNA)	
Normal	20 (91)
Beginning of malnutrition	1 (4.5)
Malnutrition	1 (4.5)

*Data are presented as mean ± standard deviation or frequency and percentage (%).ALM/ht^2^, appendicular skeletal muscle divided by height squared; BMI, body mass index; BF%, body fat; FM, fat mass; SMI, skeletal muscle index; SMM, skeletal muscle mass; MNA, mini-nutritional assessment*.

### Dietary Intake

The habitual dietary intakes for the study participants without and with the additional one portion of fish compared with DRI values among sex groups are presented in Tables 2, 3.

**Table 2:** The habitual dietary intake of the study participants compared with DRI values among sex groups.

**Parameters**	**Men (***n** **=*** **8)****			**Women (***n** **=*** **14)****		
	**habitual intake**	**DRI**	***P*-value**	**habitual intake**	**DRI**	***P-*value**
Energy (Kcal)	1,570.2 ± 179.02	2,000	0.000	1,530.8 ± 319.74	1,600	0.244
Carbohydrates (g/d)	234.3 ± 50.57	130	0.001	208.9 ± 60.49	130	0.001
Carbohydrates (%kcal)	61.0	45–65	-	55.9	45–65	-
Protein (g/d)	60.6 ± 8.60	56	0.167	61.3 ± 13.30	46	0.001
Protein (%kcal)	15.8	10–35	-	16.4	10–35	-
Total fat (g/d)	43.4 ± 9.10	35.50	0.061	50.0 ± 22.10	35.5	0.065
Total fat (%kcal)	25.4	20–35	-	30.1	20–35	-
Saturated fat (g/d)	12.6 ± 4.00	16.7	0.062	15.8 ± 7.38	12	0.077
MUFA (g/d)	10.7 ± 6.40	17.7	0.063	12.1 ± 13.27	12.7	0.875
PUFA (g/d)	5.3 ± 3.20	13	0.063	5.8 ± 4.04	9.8	0.063
Omega-3 (g/d)	0.4 ± 0.20	1.6	0.001	0.5 ± 0.31	1.1	0.001
Vitamin D (μg/d)	1.2 ± 1.60	10	0.000	1.3 ± 2.61	10	0.000
Vitamin E (mg/d)	3.0 ± 1.80	15	0.000	2.6 ± 1.68	15	0.000

*Data are presented as mean ± standard deviation. MUFA, monounsaturated fatty acids; PUFA, polyunsaturated fatty acids*.

**Table 3:** The habitual dietary intake with the additional portion of fish (~160 g) for the study participants compared with DRI among sex groups.

	**Men (***n** **=*** **8)****				**Women (*****n** **=*** **14)**	
	**habitual intake**	**DRI**	***P*-value**	**habitual intake**	**DRI**	***P*-value**
Energy (Kcal)	1,716.4 ± 179.02	2,000	0.002	1,680.0 ± 319.74	1,600	0.561
Carbohydrates (g/d)	234.3 ± 50.57	130	0.001	208.9 ± 60.49	130	0.001
Carbohydrates (%kcal)	55.4	45–65	-	50.6	45–65	-
Protein (g/d)	90.1 ± 8.60	56	0.000	90.9 ± 13.30	46	0.000
Protein (%kcal)	21.3	10–35	-	22.0	10–35	-
Total fat (g/d)	46.6 ± 9.10	35.5	0.010	53.2 ± 22.10	35.5	0.010
Total fat (%kcal)	24.8	20–35	-	29.0	20–35	-
Saturated fat (g/d)	13.4 ± 4.00	16.7	0.355	16.6 ± 7.40	12	0.136
MUFA (g/d)	11.3 ± 6.40	17.7	0.067	12.8 ± 13.30	12.7	0.978
PUFA (g/d)	5.3 ± 3.20	13	0.081	7.0 ± 4.00	9.8	0.023
Omega3 (g/d)	1.6 ± 0.20	1.6	0.917	1.7 ± 0.30	1.1	0.000
Vitamin D (μg/d)	10.1 ± 1.60	10	0.839	10.3 ± 2.60	10	0.684
Vitamin E (mg/d)	4.3 ± 1.80	15	0.000	4.0 ± 1.70	15	0.000

*Data are presented as mean ± standard deviation. MUFA, monounsaturated fatty acids; PUFA, polyunsaturated fatty acids*.

The habitual dietary intakes for men showed a significantly lower intake of total energy, omega-3 fatty acids, vitamin D, and vitamin E than DRI values (*p* < 0.05). However, with the additional intake of one portion of fish, protein and fat intakes significantly increased above the DRI (*p* < 0.05), whereas energy intake and vitamin E remained significantly lower than DRI (*p* < 0.05). Omega-3 fatty acids and vitamin D increased and met the DRI value.

In women, a significantly lower intake of omega-3 fatty acids, vitamin D, and vitamin E in their habitual diet than DRI values (*p* < 0.05) were found. Alternatively, a significantly higher intake of protein than DRI values was noted (*p* < 0.05). The habitual dietary intake plus one portion of fish showed a significant increase in fat and omega-3 fatty acid compared with DRI values (*p* < 0.05). The protein and vitamin E intake remained significantly higher and lower, respectively, than DRI values (*p* < 0.05). Vitamin D increased and met the DRI values.

### Outcome Measures

The effects of the additional intake of one portion per day of fish twice a week for 5 and 10 weeks on the study variables are presented in Table 4. Consuming a portion of fish twice a week significantly improved the SMM and ALM/ht^2^ with a positive percentage of relative change at weeks 5 and 10 compared with those at baseline (*p* < 0.05). The waist circumference and waist–hip ratio significantly decreased at weeks 5 and 10 compared with those at baseline (*p* < 0.05). A significant decrease in BF% and FM was also noted at weeks 5 and 10 compared with those at baseline (*p* < 0.05). Handgrip strength at week 10 significantly increased compared with that at baseline (*p* < 0.05). In addition, a significant decrease in gait speed <0.8 m/s was observed at weeks 5 and 10 compared with that at baseline. Alternatively, all study variables did not significantly change among time points.

**Table 4:** Relative changes at weeks 5 and 10 associated with the additional intake of one portion (150–170 g) of fish per day in the study participants.

***N =* 22**	**Baseline**	**Week 5**	**Week 10**	**Relative change from baseline (%)**	
				**Week 5**	**Week 10**
Body weight (kg)	64.25 ± 11.71	64.17 ± 11.72	63.89 ± 11.47	−0.12	−0.56
BMI (kg/m^2^)	25.97 ±4.82	25.99 ± 4.93	25.88 ± 4.87	0.07	−0.34
Waist (cm)	90.50 ± 10.15	87.83 ± 9.83	86.72 ± 6.89	−2.99^*^	−4.17^**^
Hip (cm)	102.28 ± 8.60	100.56 ± 7.53	100.44 ± 7.85	– 1.68	– 1.79
Waist–hip ratio	0.89 ± 0.09	0.88 ± 0.10	0.86 ± 0.08	−1.12^*^	−3.37^*^
BF %	39.16 ± 9.22	37.22 ± 11.26	37.14 ± 11.04	−4.95^*^	−5.15^*^
FM (kg)	25.81 ± 8.96	24.80 ± 9.67	24.64 ± 9.53	−3.91^*^	−4.53^*^
SMM (kg)	20.62 ± 3.87	21.14 ± 3.81	21.07 ± 3.67	2.52^*^	2.18^*^
SMI (%)	31.21 ± 4.68	31.84 ± 5.99	31.82 ± 5.75	2.01	2.00
ALM/ht^2^ (kg/m^2^)	6.05 ± 1.02	6.19 ± 1.01	6.21 ± 0.98	2.31^*^	2.64^*^
Handgrip strength (kg)	20.63 ± 7.11	20.82 ± 8.65	21.86 ± 9.11	1.00	5.96^*^
Gait speed <0.8 m/s	1.35 ± 0.78	1.13 ± 0.67	0.97 ± 0.43	−16.29^*^	−28.14^*^

*Data are presented as mean ± standard deviation*.

* *P < 0.05 significantly different from the baseline;*

** *P < 0.01 significantly different from the baseline. ALM/ht^2^, appendicular skeletal muscle divided by height squared; BMI, body mass index; BF%, body fat; FM, fat mass; SMI, skeletal muscle index; SMM, skeletal muscle mass*.

## Discussion

Our findings demonstrated that consuming fish twice a week for 5 and 10 weeks significantly enhanced the SMM, ALM/h^2^, and gait speed <0.8 m/s compared with those at baseline. In addition, handgrip strength significantly improved at week 10 compared with that at baseline. Consuming fish at least twice a week is recommended by the American Heart Association as part of a healthy diet (30). It has been reported that fish intake has, without doubt, a vital role in maintaining muscle mass (31). The mechanism behind the beneficial impacts of fish on muscle mass and function is likely multifactorial; including mechanisms of certain nutrients that fish has composed.

Protein provides essential amino acids that stimulate muscle protein synthesis (8). Adequate intake of protein can prevent skeletal muscle atrophy, impaired muscle growth, and functional decline (32). A growing body of evidence suggests that older adults need a higher protein intake and shows that higher protein intake is favorable in maintaining their health and functionality (33–35). The main concern for older age is that the anabolic response to protein intake might be blunted; thus, increasing their intake of protein can help sustain nitrogen balance and prevent loss of muscle mass and function (14, 35). In this study, the addition of fish to the participants' normal diet led to an increase in protein intake above the DRI values for both men and women, which might help enhance protein synthesis, improving muscle mass and function. Higher intake of animal-protein foods (i.e., meat, fish, and eggs) was associated with the preservation of muscle mass and function among older adults (36). Although no interventional study has been conducted on humans to assess the effects of fish protein alone on muscles, a study on rats has shown that fish protein intake increases skeletal muscle weight (37). A previous cross-sectional study has reported an increase in handgrip strength of 0.43 kg in men and 0.48 kg in women for each additional portion of fatty fish consumed per week (38).

Vitamin D deficiency is highly prevalent in Saudi Arabia across all demographics and considered as a public health concern in the country (39). Consuming foods rich in vitamin D, such as fish, should be encouraged (20). In the current study, intake of vitamin D was below the DRI values; however, with the additional portion of fish, vitamin D levels increased and met the DRI values. Vitamin D has been identified as necessary for normal development and growth of muscle fibers, with its deficiency adversely influencing muscle function (40). Food consumption combined protein and other essential nutrients for the maintenance of muscle mass including vitamin D (i.e., fish) may improve muscle mass and function more effectively. A study involving older adults with sarcopenia has shown that the combined supplementation of whey protein and vitamins D and E significantly enhances the relative SMI and muscle strength (41). Bauer et al. have reported that a vitamin D oral supplementation with leucine-enriched whey protein improves muscle mass and lower-extremity function in older adults (42).

Fish is also an important source of omega-3 fatty acids (43), and particular attention has been focused on the role of omega-3 fatty acids on muscle mass and function. Our omega-3 fatty acids findings conform to previous studies wherein dietary supplementation with fish omega-3 fatty acids improves muscle protein degradation and increases muscle strength in older adults (38, 44). It has been reported that adding 4 g/day of fish omega-3 fatty acids for 8 weeks to the normal diet of older adults increased the acute amino acid-induced activation of the mTOR-p70s6k signaling pathway and muscle protein synthesis (45). Omega-3 fatty acids can diminish muscle decline by increasing the functional capacity by growing the intracellular metabolic signal in older adults (46, 47). Evidence shows that inflammation could play a role in the genesis of sarcopenia (48) and the anti-inflammatory actions of omega-3 fatty acids may also play a role in preventing sarcopenia (38). Intake of sufficient amount of omega-3 fatty acids could represent an effective nutritional therapy for individuals with sarcopenia (20).

Although vitamin E remained below the DRI values even after adding the fish portion, it might be that the relatively small increase in its level in combination with the increase in omega-3 intake during the intervention improved muscle mass and function more effectively. The association between vitamin E and inflammation has gained popularity recently. Vitamin E has antioxidative capacity, and supplementation of vitamin E was shown to protect against oxidative stress and inflammation (49). A study by Meydani et al. (50) has found that vitamin E supplementation lowers the expression of oxidative stress markers following a downhill run among adults.

No significant differences were observed between the outcome measurements between weeks 5 and 10, however, frequent fish consumption seems to improve sarcopenia parameters. It should be mentioned that the available data in the literature are inadequate to determine whether all types of fish, as a food, have the same beneficial impacts on muscles. More observational and intervention studies are warranted in this area. In this study, no significant differences in body weight were observed. One portion of fish provided an additional 145–165 kcal, a relatively minor contribution to the daily intake. Significant decreases in FM, BF%, waist circumference, and waist–hip ratio were observed at weeks 5 and 10 compared with those at baseline. Arciero et al. (51) have reported that consuming high-protein meals more frequently (6 × /day) decreased abdominal fat and increased muscle mass.

In the current study, although all participants reported that their body weight was stable in the last three months, the self-reported energy intakes for men were significantly lower than DRI values. Under-reporting of daily energy intake is a common and acknowledged source of measurement error in the assessment of food intake (52, 53). It has been reported that older adults under-report more than younger populations (54) and men in our study were older than the women.

It should be mentioned that the sample size was not calculated to detect differences by sex. The effects of sex on the outcome measures should be considered in future nutritional intervention studies. One of the strengths of this study is the area of research itself. Further, to the best of our knowledge, this is the first intervention study that evaluated the beneficial impact of fish as a food on sarcopenia parameters. Nonetheless, this study has some limitations that should be considered when interpreting its results. The primary limitation was the lack of a control group due to the lack of responses to the study announcement and this limits the strength of our findings. Also, the study participants were free-living, and we did not control their dietary intakes and physical activity, even though they were instructed to maintain their habitual diet and physical activity during the intervention period. In addition, middle-age and older adult participants were included in this study which may affect the homogeneity in the participants' age. The relatively small sample size was also acknowledged. Finally, this study only involved independently living participants thus, the beneficial impact of fish consumption on older adults who have an instant need for keeping mobility and physical function are warranted.

In conclusion, this study shows that consuming fish (i.e., red sea bass) twice a week for 10 weeks could improve sarcopenia parameters and muscle mass and function in middle-age and older men and women. These findings suggest that fish have an important role on muscle health by different mechanisms, including effects on muscle protein synthesis, inflammation, and oxidative stress. Intakes of protein, vitamin D, omega-3 fatty acids, and antioxidants could all be crucial for optimal muscle function and may slow the progression of sarcopenia. Further well-designed interventional studies with fish intake among older adults are recommended to confirm the beneficial impact of fish on muscle health.

## Data Availability Statement

The original contributions presented in the study are included in the article/supplementary material, further inquiries can be directed to the corresponding author/s.

## Ethics Statement

Informed consent was obtained from all participants. The study was approved by the Ethics Committee of the Institutional Review Board at King Saud University (Reference No. 19-0834/ IRB). All procedures were conducted according to the Declaration of Helsinki. The patients/participants provided their written informed consent to participate in this study.

## Author Contributions

MHA and MMA: conceptualization and data interpretation. MMA: investigation and data analysis. MHA: visualization, supervision, writing the original draft of the manuscript, and review and editing. Both authors have read and approved the final manuscript.

## Funding

Researchers Supporting Project Number (RSP-2021/338), King Saud University, Riyadh, Saudi Arabia.

## Conflict of Interest

The authors declare that the research was conducted in the absence of any commercial or financial relationships that could be construed as a potential conflict of interest.

## Publisher's Note

All claims expressed in this article are solely those of the authors and do not necessarily represent those of their affiliated organizations, or those of the publisher, the editors and the reviewers. Any product that may be evaluated in this article, or claim that may be made by its manufacturer, is not guaranteed or endorsed by the publisher.
